# Standardization of flow cytometry and cell sorting to enable a transcriptomic analysis in a multi-site sarcoidosis study

**DOI:** 10.1371/journal.pone.0281210

**Published:** 2023-03-09

**Authors:** Roman E. Magallon, Laura D. Harmacek, Nicholas K. Arger, Pineet Grewal, Linda Powers, Brenda R. Werner, Briana Q. Barkes, Li Li, Kristyn MacPhail, May Gillespie, Elizabeth K. White, Sarah E. Collins, Talyor Brown, Jessica Cardenas, Edward S. Chen, Lisa A. Maier, Sonia M. Leach, Nabeel Y. Hamzeh, Laura L. Koth, Brian P. O’Connor

**Affiliations:** 1 Center for Genes, Environment, & Health, National Jewish Health, Denver, Colorado, United States of America; 2 Department of Medicine, University of California, San Francisco, San Francisco, California, United States of America; 3 Department of Medicine, University of Iowa, Iowa City, Iowa, United States of America; 4 Division of Environmental and Occupational Health Sciences, National Jewish Health, Denver, Colorado, United States of America; 5 Department of Immunology and Genomic Medicine, National Jewish Health, Denver, Colorado, United States of America; 6 Division of Pulmonary and Critical Care Medicine, Baltimore, Maryland, United States of America; The University of Texas MD Anderson Cancer Center, UNITED STATES

## Abstract

The contribution and regulation of various CD4^+^ T cell lineages that occur with remitting vs progressive courses in sarcoidosis are poorly understood. We developed a multiparameter flow cytometry panel to sort these CD4^+^ T cell lineages followed by measurement of their functional potential using RNA-sequencing analysis at six-month intervals across multiple study sites. To obtain good quality RNA for sequencing, we relied on chemokine receptor expression to identify and sort lineages. To minimize gene expression changes induced by perturbations of T cells and avoid protein denaturation caused by freeze/thaw cycles, we optimized our protocols using freshly isolated samples at each study site. To accomplish this study, we had to overcome significant standardization challenges across multiple sites. Here, we detail standardization considerations for cell processing, flow staining, data acquisition, sorting parameters, and RNA quality control analysis that were performed as part of the NIH-sponsored, multi-center study, BRonchoscopy at Initial sarcoidosis diagnosis Targeting longitudinal Endpoints (BRITE). After several rounds of iterative optimization, we identified the following aspects as critical for successful standardization: 1) alignment of PMT voltages across sites using CS&T/rainbow bead technology; 2) a single template created in the cytometer program that was used by all sites to gate cell populations during data acquisition and cell sorting; 3) use of standardized lyophilized flow cytometry staining cocktails to reduce technical error during processing; 4) development and implementation of a standardized Manual of Procedures. After standardization of cell sorting, we were able to determine the minimum number of sorted cells necessary for next generation sequencing through analysis of RNA quality and quantity from sorted T cell populations. Overall, we found that implementing a multi-parameter cell sorting with RNA-seq analysis clinical study across multiple study sites requires iteratively tested standardized procedures to ensure comparable and high-quality results.

## Introduction

Sarcoidosis is a systemic, granulomatous disease of unknown etiology, characterized by heterogeneous severity, onset and clinical disease course [1]. Studies have characterized many different T cell effector lineages which may affect the pathobiology of sarcoidosis including Th1 T cell skewing [2, 3] and IFN*γ* production [4] in the CD4+ T cell compartment. We have also demonstrated a role for the Th17 CD4+ T cell lineage and related Th17.1 cells in driving sarcoidosis disease progression [5]. While these lymphocyte subtypes have been implicated, it is unclear how these lineages contribute to disease course over time. To examine this immunologic relationship between CD4+ T cells and clinical course, we focused on longitudinal changes in CD4+ T cell subsets and their relationship to disease phenotype in patients recently diagnosed with pulmonary sarcoidosis. Our current NIH-sponsored clinical research study (R01HL136681) known as BRonchoscopy at Initial sarcoidosis diagnosis Targeting longitudinal Endpoints (BRITE), utilizes a longitudinal, multi-center, flow cytometry-based study structure. The BRITE study centers include the University of California San Francisco, National Jewish Health, University of Iowa, and Johns Hopkins Medical Institute. This multi-site design enhances the study’s ability to achieve the targeted recruitment numbers, enhance geographic generalizability of study findings, promote a diverse subject population, include a range of disease phenotypes, and reduce the effect of unplanned challenges (eg. the COVID-19 pandemic). However, comparing data across multiple study sites is very challenging due to variability within the human subject population and the experimental instruments specific to each location. While our recent publication provides a detailed description of the overall clinical research design of BRITE, discussion of the critical flow cytometry sorting standardization required for multi-site studies was outside the scope of that manuscript [6].

Multicolor flow cytometry is a versatile technology that allows simultaneous analysis of hundreds of thousands of single cells that comprise multiple subpopulations. Moreover, these subpopulations of interest can be sorted into pure populations for downstream experimental applications [7] if the proper controls are implemented [8]. Many diseases are evaluated using flow cytometry analysis, such as T cell subset examination, and this requires standardized methodology. Thus, multiparametric flow cytometry is a uniquely useful method to obtain immunophenotyping that can be used for analysis, diagnostics, and monitoring, such as with sarcoidosis clinical course over time. Moreover, flow cytometry sorting of specific immune cell populations enables downstream molecular analysis, such as mRNA sequencing (RNA-seq). Our group has experience optimizing flow cytometry and NGS procedures for immune cell analysis [9–13].

While there are advantages to multi-site study designs, especially for rare diseases with a diverse and complex etiology and/or course, there are also significant technical and standardization challenges when trying to employ flow cytometry sorting with downstream NGS analysis. If not properly addressed, these technical challenges will lead to inter-site data compatibility problems that significantly reduce the potential impact of the overall study. Clinical trials have pioneered a path to mitigate these challenges via a flow standardization strategy, particularly during drug development due to the stringent regulatory environment [14–16]. Additionally, consortiums including the EuroFlow and FrenchFlow groups have implemented similar stringent strategies and procedures to meet this need with reproducible results mainly focusing on blood cancers, both for clinical and research purposes [17–19]. There are also comprehensive technical guidelines that detail protocols for flow cytometry and cell sorting [20]. While these studies provide a framework to standardize quantitative cytometric analysis, there are few reports examining strategies to deal with the enhanced challenges of multicenter studies utilizing flow cytometry sorting of cells for downstream RNA-seq analysis. Human immunophenotying by flow cytometry requires difficult standardization procedures to account for population variation and/or multi-site differences [21]. Studies that incorporate flow cytometry sorting of immune cell populations to enable downstream applications such as RNA-seq, have an increased technical burden due to the requirement for high quality nucleic acid material. Therefore, our BRITE study was designed to generate high quality data across the multi-site structure to enable accurate data comparisons and analysis of both the flow cytometry and NGS data sets.

During the study design period, we recognized the challenges of conducing a flow cytometry sorting with RNA-seq analysis plan at four different sites due to the potential for variability during cytometry procedures. For this study, we took an integrated approach by coordinating the resources of four active centers of sarcoidosis research and clinical practice. We optimized an interlaboratory, cross-machine standardization of a 10-color flow panel across four cytometry laboratories. We applied strict adherence to standard operation procedures for the collection, processing, and analysis of patient samples. Herein, we summarize the methodology to achieve reproducible results from geographically diverse institutions across the United States.

## Materials and methods

For the first round of procedural and flow cytometry optimization, PBMCs were collected in EDTA tubes from a healthy subject and cryopreserved in 10% dimethylsulfoxide (DMSO) in heat-inactivated fetal bovine serum (FBS) (Thermo Fischer Scientific) and stored in liquid nitrogen. Vials of these frozen PBMCs were sent by the coordinating laboratory to each participating laboratory. This first round of optimization utilized single stain antibodies with manual mixing of staining cocktails by each site. Once the optimal staining panel was verified, the antibody mixture was advanced to premixed, lyophilized cocktails produced by the manufacturer. For the second round of optimization, fresh EDTA PBMC samples from healthy subjects were isolated using ficoll and LeucoSep tubes locally at each participating laboratory and processed for staining immediately upon collection.

### Lyophilized cocktails

To minimize batch to batch variability across sites during sample processing, we used a lyophilized antibody cocktail consisting of eight premixed antibodies that was fabricated and lyophilized for the flow cytometry staining panel used in this study (BioLegend, San Diego, CA). Because the final panel consisted of three antibodies containing brilliant violet technology, one of these antibodies was added in the appropriate concentration after the lyophilized cocktail was resuspended with appropriate stabilization buffers (BD Biosciences, Franklin Lake, NJ). We included a live/dead stain to allow gating on live cells in the analysis. The composition of the lyophilized cocktail was as follows: CD4 (clone OKT4) FITC, CD3 (clone SK7) Alexa Fluor700, CD45RA (clone HI100) APC/Fire760, CD127 (clone A019D6) PE, CD25 (clone BC96) PE/Cyaine7, CD194/CCR4 (cloneL291H4) APC, CD183/CXCR3 (clone G026H7) Brilliant Violet 421, CD45RO (clone UCHL1) Brilliant Violet 711, CD196 (CCR6) (clone G03E3) PE/Dazzle 594.

We also designed a separate lyophilized cocktail that we included with each sample to serve as the fluorescence minus one (FMO) control for CCR4, CCR6 and CXCR3. This cocktail included all antibodies listed above except these three chemokine receptors. The appropriate concentration of each antibody (minus one) was added to each FMO control using separate liquid reagents for CCR4, CCR6 and CXCR3.

### Live/dead stain

Live/dead was titrated to achieve the best staining index, which was 1:500 for 20 minutes in PBS, and then quenched with two washes of FACS buffer (BD Biosciences, Franklin Lake, NJ); Other concentrations tested were 1:100, 1:300, and 1:1000. The lot of Zombie Aqua live/dead stain (Biolegend, San Diego, CA) was coordinated across study sites.

### Microfluorescent standardization beads

All lasers were matched to CS&T Research Beads (BD Biosciences, Lake Franklin, NJ)—Catalog# 655051 (3 tubes each); Tube Lot# #93017. The Flow-validated lot (AK04) of Rainbow Calibration Particles, 8 peaks (Spherotech, Lake Forest, IL); (referred hereafter in this manuscript to as Rainbow beads) were also used as a secondary laser calibration in all participating laboratories throughout the study.

### Tracking flow analysis performance over time

Tracking the potential variation of a flow panel’s performance over time in the context of a study site’s cytometer is another important optimization consideration for longitudinal studies. We monitored cytometer variation by including a site-specific biological control sample to our analysis protocol that was analyzed at regular intervals during the study period. Our site-specific biological control sample met the following requirements: (1) it contained cells that expressed the targeted molecular epitopes used by the lyophilized panel to enable testing and tracking of population resolution, and (2) sufficient quantity of the sample was collected and frozen down into single use vials in 10% DMSO in FBS so that it could be included for periodic testing throughout the duration of the study (with approximately 60% live cell recovery rates from thawed cells). The site-specific biological control was designed to be used in the following scenarios: 1) approximately once every three months for periodic assessment, 2) after a laser reconfiguration or service of the flow cytometer, and 3) use of a new lot of antibody or lyophilized panel. Data from the site-specific control runs could then be analyzed to assess population resolution as well as population enumeration compared to previous analyses. Any significant deviations in these metrics indicated a site-specific processing change that would need to be addressed promptly. Thus, we incorporated a routine analysis of the biological controls in our BRITE study methodology to enable identification and correction of potential site-specific flow cytometry variance that can occur over the course of a longitudinal study.

### mRNA isolation

Sorted T cell subsets were stored in Qiagen RLT buffer +1% β-mercaptoEthanol (B-ME) to maintain the quality and quantity of RNA. Standard Qiagen (Germantown, MD) protocols for the QiaShredder (#79656) and RNeasy Mini (#74104) kits were then followed to extract the RNA.

### Ethics approval statement

This study involves human participants and was approved by an Ethics Committee(s) or Institutional Board(s). Each participating study site obtained approval by the Institutional Review Boards at each center. This study will be conducted in accordance with globally accepted standards of good practice The study protocol has been approved by the Institutional Review Boards at each center: National Jewish Hospital (IRB# HS-3118), University of Iowa (IRB# 201801750), Johns Hopkins University (IRB# 00149513), and University of California, San Francisco (IRB# 17–23432). Written informed consent was obtained on all participants enrolled in this study.

## Results

### Overall strategy

**Fig 1:**
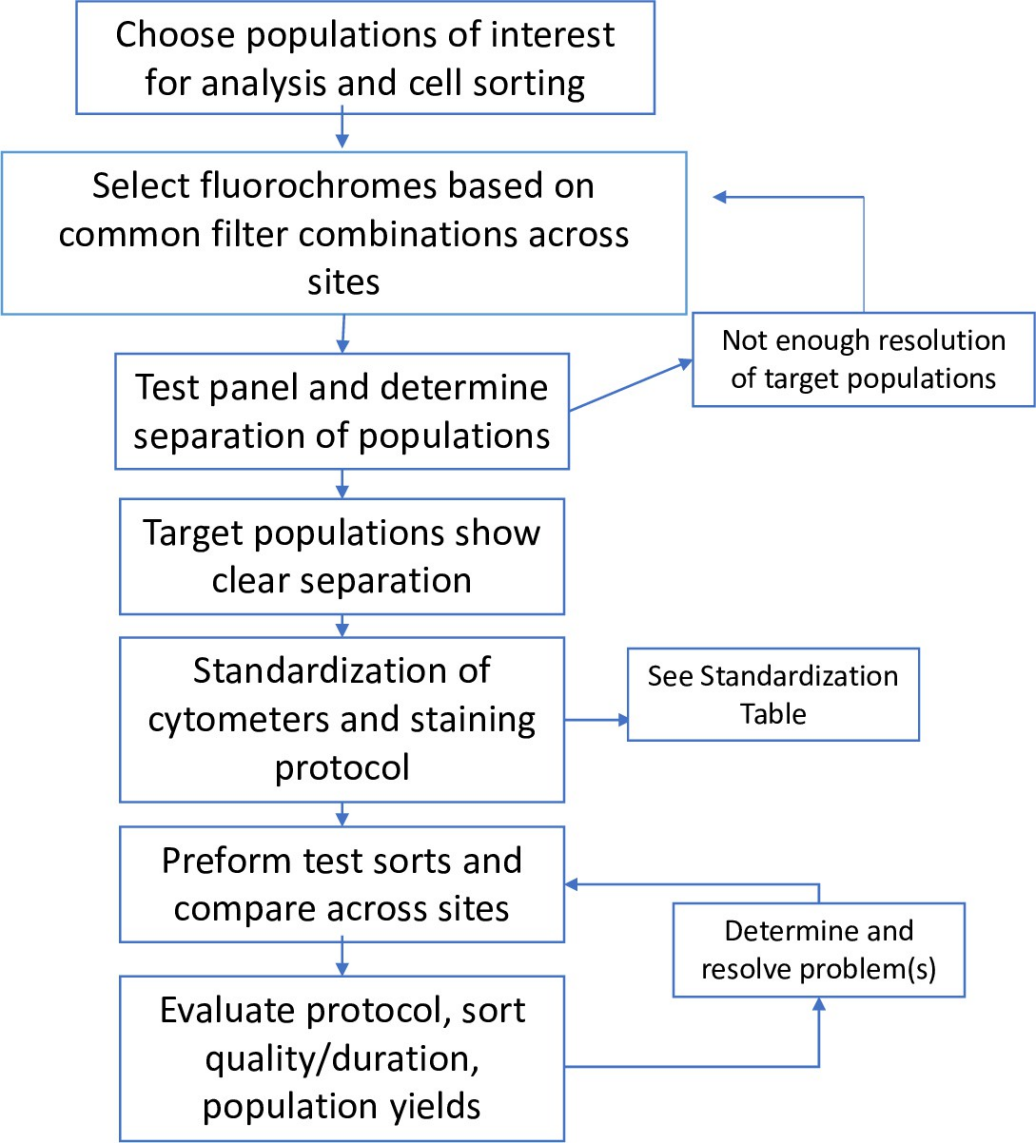
Overall schematic for flow cytometry optimization and standardization across clinical study sites. Schematic outlining the strategy used to improve the resolution of targeted populations of interest via flow cytometry.

Flow cytometry studies examining methodology choices have demonstrated that a centralized analysis center strategy vs an individual manual gating strategy can result in similar types of data variation [22]. However, this complexity is increased for multi-site immune cell sorting studies due to the additional challenges and variability of real time population isolation using multiple different cytometers at various locations. Finally, the requirements of downstream applications such as RNA-seq with the sorted immune populations further increase the requirements for stringent optimization and standardization between study sties. Taking this complexity into consideration for the BRITE multi-site study, we conceptualized several approaches for an optimal method to obtain pure cell populations of interest for immunophenotying and subsequent RNA-seq analysis. We considered three different approaches to address the complexity of biospecimen analysis in a multi-site clinical study. These approaches were: 1) shipping blood to a central site for PBMC processing followed by flow cytometry analysis and sorting; 2) shipping frozen, isolated PBMCs in 10% DMSO in FBS to a central site for subsequent processing and flow cytometry sorting; or 3) processing and performing flow cytometry analysis at each site using freshly isolated cells. For each of these strategies, sorted cell populations would be maintained in an RNA stabilizer (ex. Qiagen RLT buffer) and kept frozen until RNA isolation. We reasoned that while central cell flow sorting and analysis is a common practice, we required robust cell surface protein detection to enable accurate identification of cell populations for sorting and downstream RNA-seq analysis, which can be affected by shipping and/or freeze/thaw cycles. Based on our prior experience staining chemokine receptors, we concluded that option 3, performing flow cytometry analysis and sorting with freshly isolated human samples would enable the optimal detection of the chemokine receptors, and maximize the success of the BRITE study goals. Given these priorities, we identified multiple steps that were essential for standardization of multi-parameter flow cytometry and flow sorting of CD4+ T cells across multiple research facilities. These steps are: (a) Panel design and antibody reagent selection, taking into account varying laser configurations across each facility, (b) flow cytometer voltage standardization, (c) laser performance over time, and (d) RNA quality control analysis. **Fig 1** and **Table 1** display the sequential steps we followed.

**Table 1:** Standardization actions taken and purpose for the identification and collection of cell populations in flow cytometry.

*Action*	*Purpose*
CS&T Beads	Standardize cytometer settings: PMT Voltages, laser delays, Quality Control
Rainbow beads	Standardize the compensation matrix
Lyophilized Cocktail for staining	Standardize Antibody Concentrations and FMO Controls to reduce variation across sites
Coordinate Essential Reagents	Use of identical lots and/or catalog numbers for critical reagents to reduce variation
Detailed Standard Operating Procedures Manual	Standardize sample handling, processing and reagents
Site Specific Control (Frozen PBMCs)	Assess variation in performance of site cytometers over time
Gating Strategy	Ensure sorting standardization of target populations

### Panel design

**Fig 2:**
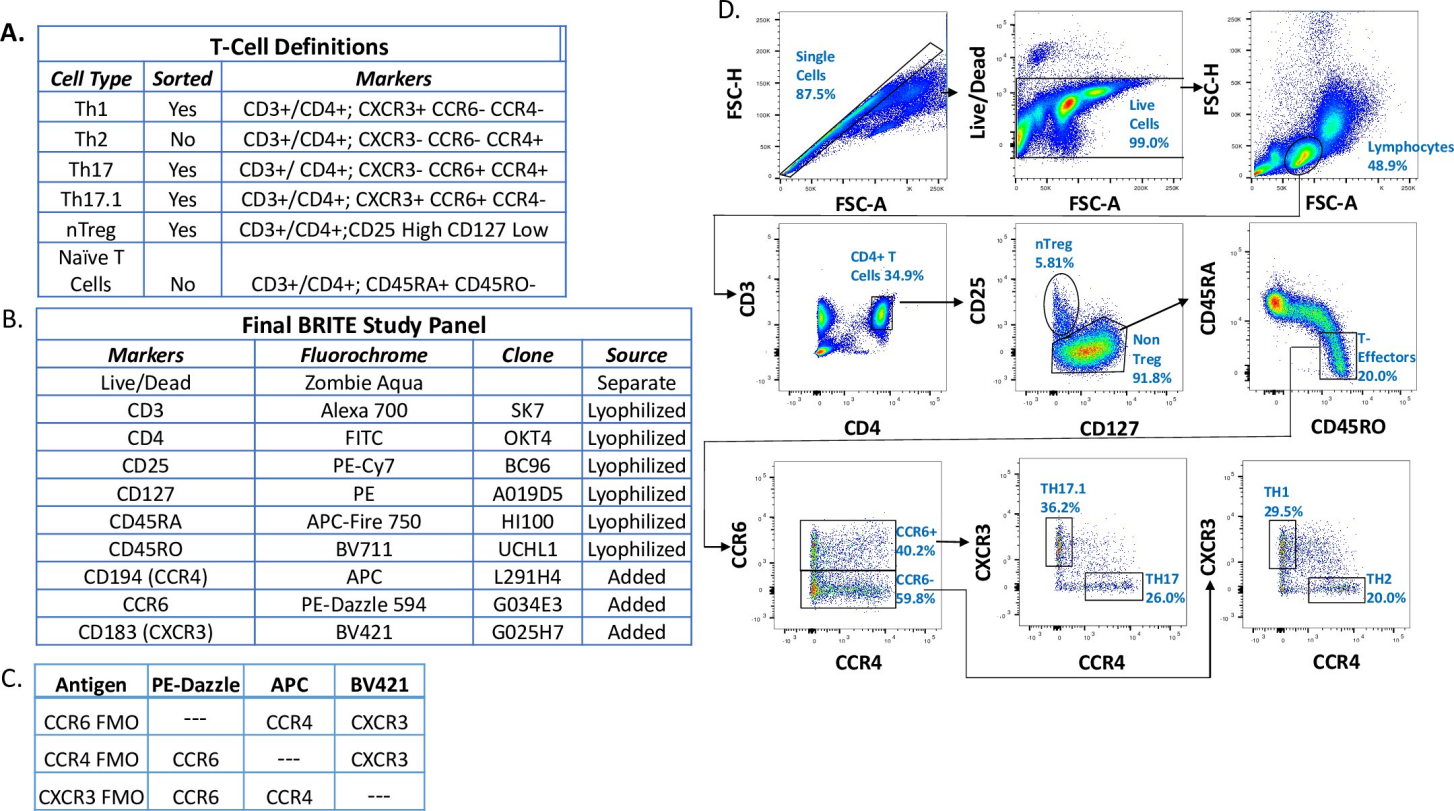
Gating strategy used to differentiate immune cell populations based on cell surface markers. A) Cell surface marker combinations used to identify CD4+ T cell subset populations. B) The final optimized flow panel for separation of cell populations across different sites noting which reagents were in the lyophilized antibody cocktail, added to the antibody cocktail, or stained separately. C) Fluorescence Minus One (FMO) design and addition of antibodies after reconstituting the FMO lyophilized cocktail. D) Gating strategy illustrating strategy for separation of cell populations. Note, in some plots, can’t see the gates because the blue color blends in with the populations; could consider changing the color of the gate for all plots.

The first step of the flow sort study design was careful consideration of the immune cell populations for which we planned to analyze and purify by cell sorting. Based upon prior literature in sarcoidosis [23–27] and results from our prior studies [5, 28, 29], we hypothesized that the dynamic relationship of T cell lineages (Th1, Th2, naïve T cells, Memory T cells, Th17, Th17.1, Treg cells) affects the variable pathobiology and outcomes characteristic of the sarcoidosis disease course. To test this hypothesis, we focused on achieving accurate resolution of the CD4+ T cell lineages. We followed best practices guidelines for antibody panel design strategy as described in excellent resources [30]. Based on these guidelines, we chose the epitopes summarized in **Fig 2A** to define the T cell populations of interest. Our cytometers had four channels for sorting and we chose CD4+ T cell subpopulations that we were most interested in studying based on our prior work (Th1, Th17, Th17.1, and Treg). After choosing the epitopes, we then considered the appropriate combination of fluorochrome and antibody epitope for the given cytometer laser configurations, which differed across the BRITE study sites.

**Fig 3:**
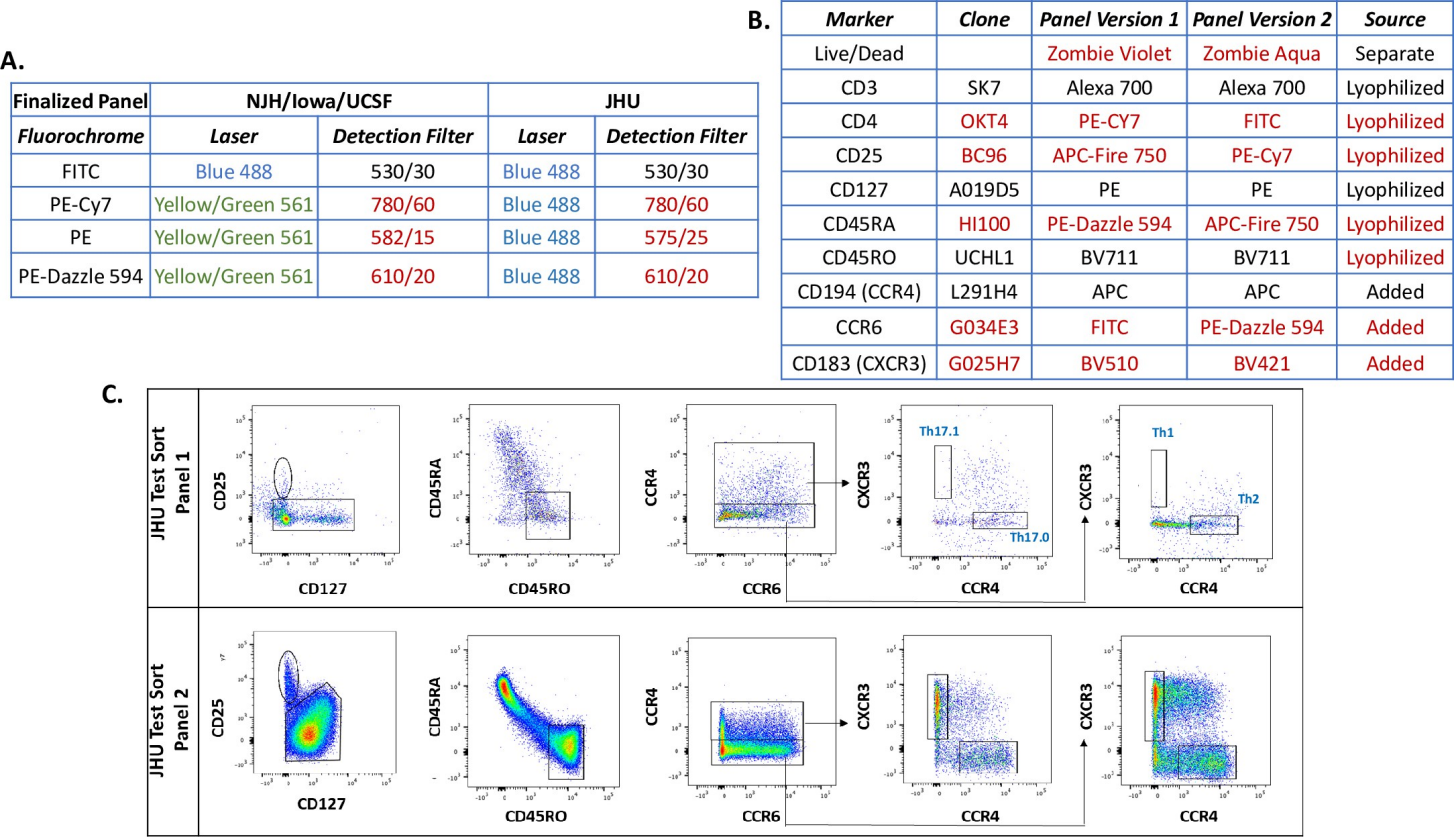
Testing of flow panels for optimal resolution of T cell subset identification due to variations in cytometer specifications between different study sites. A) Differences in cytometer laser/filter set combinations by site for the finalized panel. Red text denotes lasers/filters that differ between sites. B) Two variations of the flow panel that were tested to optimize population separation based on differences between cytometers, noting which reagents were in the lyophilized antibody cocktail, added to the antibody cocktail, or stained separately. Red text indicates fluorochromes that differ between the two panels. C) Side by side comparison of T cell subset identification by panel 1 or 2.

The optimization of different laser configurations for the different cytometers at multiple clinical sites is a significant challenge for flow cytometry studies [31]. All four BRITE sites utilized the same cytometer model: the BD FACS Aria. Three of the four sites had similar laser configurations, comprising Red, Yellow/Green, Blue and UV wavelengths. However, one research site (JHU) had access to an Aria with only three lasers that lacked the 561 laser (yellow/green). Therefore, this difference in laser number had to be incorporated into our panel design. **Fig 3A** illustrates how the 488 laser (blue) was used to excite both FITC and PE, and well as other PE derivative dyes at JHU. Thus, due to the different laser configurations, we had to design and test several varying combinations of fluorochrome and antibody epitopes in an iterative process to identify a unified, optimized panel that would work for the flow cytometers at each site.

We also considered issues specific to cellular composition and antibody fluorochrome chemistry in our optimization strategy. The expression of cell surface epitopes ranges from on/off to a spectrum of densities on a given cell, resulting in variable protein detection by flow cytometry. Another source of variable detection to be considered is the inherent intensities of fluorochromes, which are different. Therefore, the purposeful selection of epitope/fluorochrome combinations is necessary to obtain maximum resolution of protein markers, which includes the balance of detection intensity vs reduction of spectral overlap. The emission overlap can be partially corrected through spectral compensation, which is itself affected by the detection intensity. Thus, choosing a panel for a multi-site study must take into account these complex parameters. **Fig 3B** outlines a flow panel comparison performed for the BRITE study across the different sites. Low expression chemokine receptors necessitated brighter fluorochromes; for example, the fluorochrome for CCR6 was changed from a medium bright FITC to a very bright PE-Dazzle 594. CD4 is a relatively abundant epitope, and therefore a very bright fluorochrome was not necessary for detection, and thus, FITC was chosen instead. For example, FITC was especially dim in panel version 1 on the JHU cytometer and needed to be assigned to the more abundant epitope CD4. This caused some markers to be dimmer compared to the other sites, as is evident in the comparison of the two panels in the gating strategies **(Fig 3C)**. The Treg population was not resolvable in panel 1, due to a dim CD25. Moreover, three additional populations that were being sorted were not resolvable due to a dim CXCR3 detection. After testing multiple different flow cytometry panels, the populations of interest were best defined by panel 2 (**Fig 3C,** panel 2). For example, the gating of the CD45RA^+^ and CD45RO^+^ cell population was distinct at each site, and the T-effector population was clearly distinguishable. Therefore, after these optimization steps, we arrived at the final staining panel showing the best resolution for the populations of interest (**Fig 2B)**. In summary, only three of the four sites had similar laser configurations, comprising Red, Yellow, Blue, and UV wavelengths. Despite these differences, our iterative optimization produced comparable cell population detection across the four sites to distinguish the CD4+ T cell populations of interest: Naive T cells, Regulatory T cells, and multiple T effector types (Th1, Th2, Th17 and Th17.1).

Once the final antibody-fluororchrome panel was optimized, we considered the best practices for the flow cytometry staining protocol. Lyophilization of reagents has been shown to reduce variation in at least 3 ways: (1) mis-titration or pipetting errors of reagents into the master cocktail, (2) ease of experiment setup, (3) and longevity of shelf-life of reagents [17, 18, 32]. For these reasons, we chose to create a lyophilized panel for the BRITE study, as depicted in **Fig 2B**. There were two main technical issues we had to address in the development of this master lyophilized panel. The first was the fact that the live-dead stain we used was not an antibody but a lipophilic dye that requires a different staining buffer than the antibody cocktail. Thus, this dye was not included in the master panel. Second, our panel included three antibodies that used Brilliant Violet (BV) chemistry. It is not recommended to lyophilize more than two BV dyes together because of polymerization of the compounds. Therefore, we lyophilized two BV antibodies into the master panel along with other non-BV dyes, and then added the third BV antibody after the lyophilized cocktail was reconstituted with appropriate stabilizing buffers.

To allow for the most accurate gating of the T cell populations of interest, we performed FMO controls. Our panel design relies on discrimination of CD4+ T cell lineage subsets through the use of chemokine receptor expression. These receptors show a range of expression that is continuous which causes difficulty when trying to differentiate cells with intrinsic low expression versus no expression. Therefore, we designed a lyophilized FMO mixture containing the full panel of antibodies except for the three chemokine receptors of interest, CCR4, CXCR3, and CCR6. We then added the appropriate chemokine receptor antibodies after reconstitution of the FMO panel into each FMO control (**Fig 2C**). This second lyophilized FMO panel was also manufactured to enhance standardization across the BRITE study sites for FMO-based accurate gating of Treg, Th1, Th17, and Th17.1 cells. In conclusion, four methods were used to standardize panel design and laser variation across the sites.

### Flow cytometer parameters standardization

Variation in flow cytometer parameters, such as changes in laser performance over time, represents another important consideration for optimization and standardization across research study sites. Flow cytometers with the best consistency and resolution show strong excitation of fluorochromes, optimal transmission of photon emission, and efficient collection of this emission at the detector (photomultiplier tube (PMT) [33]. Cytometer settings that change over time include photomultiplier tube (PMT) voltages, which tend to decline as lasers age, and intrinsic laser delays [34]. Fluorescent microbeads alleviate several problems by allowing voltage and laser delays to be standardized [33]. We relied first on CS&T beads to measure detector performance and sensitivity on a given cytometer. These beads are a suspension of fluorospheres with a uniform size and fluorescence intensity. We took advantage of these bead’s properties to adjust the mean fluorescence intensities (MFI) for each detector in our panel (Table 2).

**Table 2:** MFI values for each detector channel.

Zombie-Aqua Live-Dead	31429
AF700 CD3	12355
FITC CD4	7071
PE-Cy7 CD25	18378
PE CD127	3242
APC-Fire750 CD45RA	46111
BV711 CD45RO	7457
APC CCR4 Red	21953
PE-Dazzle CCR6	9716
BV421 CXCR3	7253

**Fig 4:**
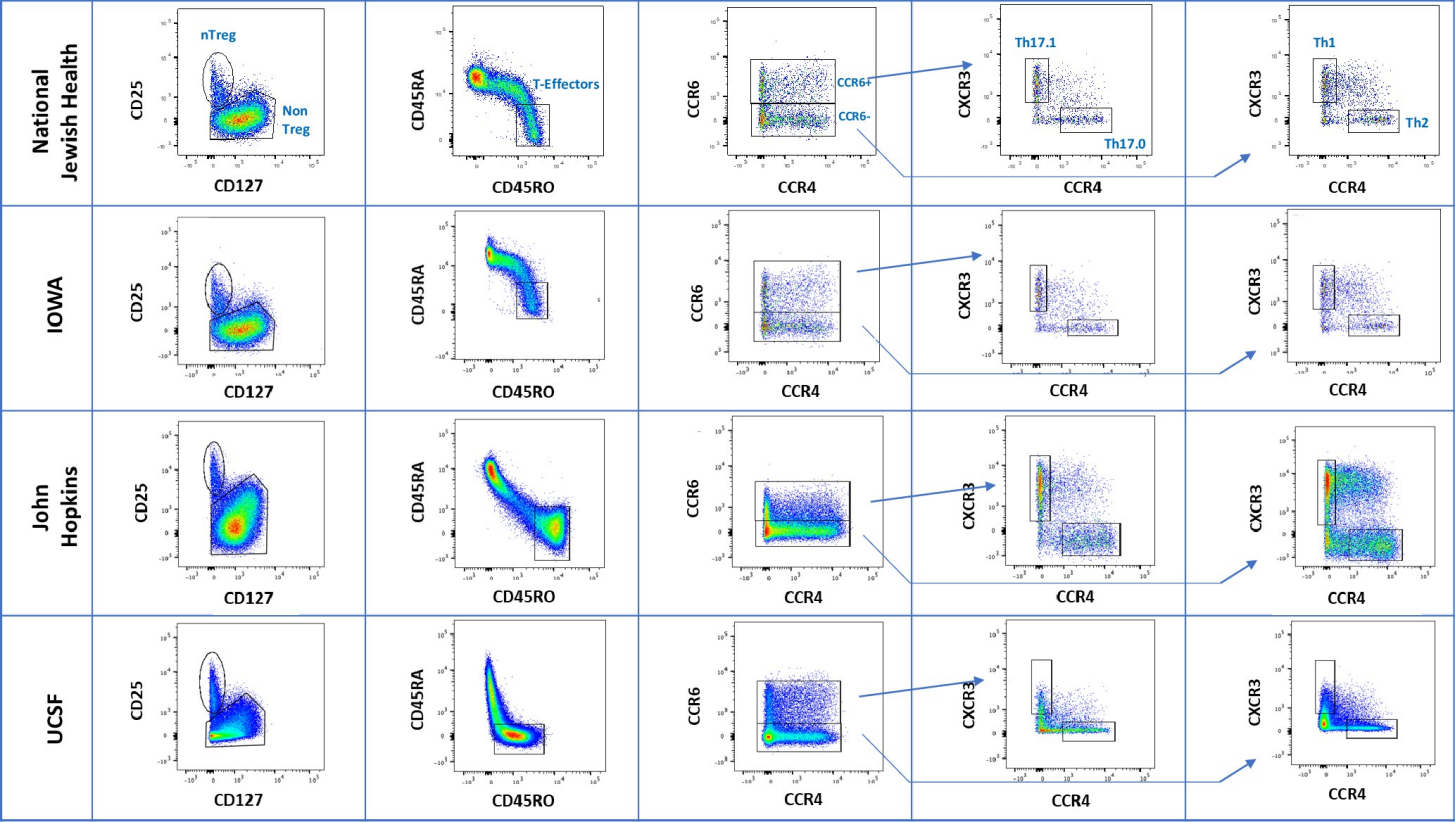
Comparison of human PBMC sample analysis between institutions after standardization. PBMCs from different human subjects were isolated, stained, and run on the flow cytometer at each study site. Shown are cell populations after gating on singlets, live/dead, lymphocytes each by FSC/SSC, and CD3+ cells. T cell subsets identified by the analysis include T effector, Th17.1, Th17, Th1, and Treg.

This process was performed at one site and the MFI settings were communicated to all other sites to standardize the cytometer parameters (S1 and S2 Figs). To reduce potential manufacturer variations, the same CS&T bead lots were used by all sites. Additionally, Rainbow Beads (Spherotech) were chosen as a secondary readout to ensure consistent cytometer laser voltage settings. The Rainbow beads measure sensitivity and linearity of flow systems and were therefore used to fine-tune voltages at each study site [35]. After each site matched voltage settings to the same lot of CS&T beads and used rainbow beads to fine tune MFI’s for each detector, each site stained and analyzed a biological sample using the full panel of antibodies to confirm an appropriate fluorescence pattern for each antibody. The flow analysis results after the optimization steps at all sites are displayed in **Fig 4,** which demonstrates consistency in population resolution and detection. Fig 4 also illustrates the expected variation across individuals for some of the T cell populations, such as CD4 T cells expressing the CD45RA and -RO antigens. Some individuals possess more double positives CD45RA+CD45RO+ cells than double negatives and vice versa and represents normal human variation [36] and is not a technical issue of staining.

### Data acquisition

The BRITE flow cytometry objectives included enumeration of T cell lineage frequencies as well as sorting of these lineages for downstream RNA-seq analysis. Thus, we utilized a gating strategy that balanced population detection for accurate enumeration with population separation for accurate cell isolation, a necessity for RNA-seq analysis. It was especially important to determine how many cells could be collected for rare cell populations of interest in this study (e.g. Treg, Th17, or Th17.1) (**Fig 2D)**. The BRITE gating strategy begins with discrimination of singlets, followed by live cell discrimination, and then lymphocyte identification (using forward scatter height versus forward scatter area). We then used expression of CD4+ and CD3+ to define the T cell population followed by selection of T regulatory cells and T helper subsets as illustrated in **Fig 2D**. As outlined in **Fig 2A**, we capture cell frequency data for six populations, while sorting four CD4+ T cell populations for downstream T cell transcriptomic analysis. The next critical aspect of optimization was to standardize the exact gates for acquisition of data related to cell fractions and cell sorting across sites. Given that the BRITE experimental approach requires site specific cell sorting, data acquisition and analysis parameters had to be standardized to ensure comparable cell population data between the study sites. After these rounds of optimization were completed, a master-gating template was created that included 11 cell populations, ultimately resolving the 4 cell populations targeted for sorting (**Fig 2D**). The master-gating template was distributed across the BRITE research sites to standardize population identification. This file was uploaded and integrated with the cytometer software on each instrument, with a centralized oversight of the template adoption enabled via video conferencing as needed. Collectively, the standardization of the flow cytometer machine-specific operations outlined here produced consistent results across sites and also longitudinally within each site. Thus, the flow cytometry standardization practices significantly reduced instrument-to-instrument variation, which ultimately would reduce noise within the collected data and thus enable higher quality data comparisons for the entire BRITE study and broader research community.

### RNA isolation from sorted T cell subsets

**Fig 5:**
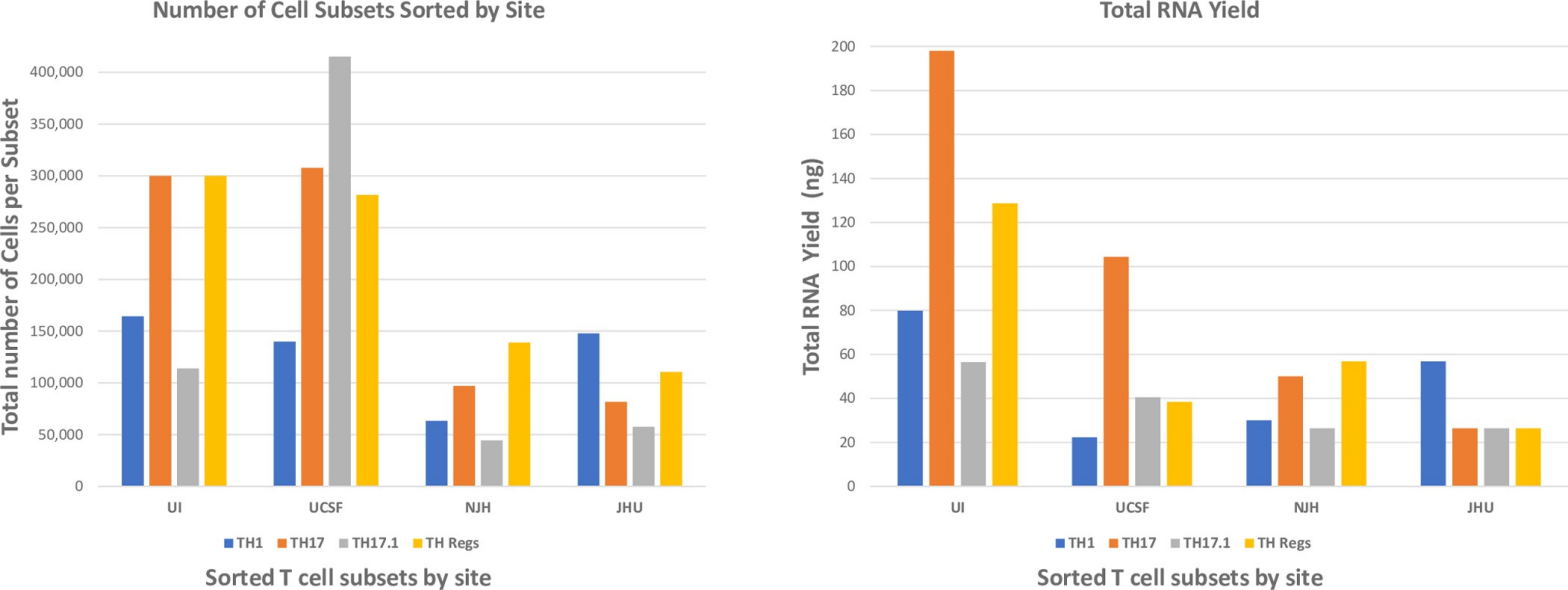
Cell number and RNA quantity obtained after cell sorting. CD4+ T cell subsets were flow sorted from PBMCs at each site using the final flow panel shown in Fig 3. Sorted populations were lysed to preserve RNA and shipped to the central laboratory where total RNA was isolated. A) Number of cells sorted in each subset by each site from one sample. There was only one sample per test, thus no error bars are added. B) Total RNA mass obtained after extractions based on number of cells sorted per site per subset.

In addition to data standardization and comparison considerations, another main design consideration of the flow cytometry sorting in the BRITE study was to enable RNA-seq transcriptional profiling of T cell subsets during the sarcoidosis disease course. Thus, determining if the standardized flow protocol could enable sorting of enough cells to isolate mRNA of sufficient quantity and quality for RNA-seq was critical to accomplishing the study goals. However, a number of factors can complicate the isolation of sufficient mRNA from sorting of multiple T cell subsets, including: 1) the natural variation in human immune cells that occurs between individuals and longitudinally within a subject; 2) variation of immune cell subsets due to disease course; and 3) variable cell transcriptional activity levels can decouple the expected mRNA yields from the number of sorted cells. Thus, we set a goal of isolating enough mRNA from sorted T cells to enable RNA-seq using a standard, low-input protocol, such as the SMARTer stranded RNA-seq kit (Takara, San Jose, CA). The sensitivity of the SMARTer kit enables RNA-seq library construction from as little as 100 pg of input RNA material. To monitor the flow sorting optimization, we tracked two factors: 1) the number of cells per subset collected during sorting at each study site, and 2) the total RNA yield from each sorted cell subset. These results are displayed in **Fig 5A** and **5B,** respectively. Our analysis revealed that while overall RNA yield was variable, as expected and discussed above, sufficient RNA was extracted to perform RNA-seq library construction from all cell subtypes using a standard, low-input methodology such as SMARTer kit. Indeed, comparing cell number versus RNA yield analysis, we concluded that for each cell subtype, 20,000 sorted cells would yield more than 1 ng of total RNA, which is above the 100 pg threshold of the SMARTer protocol. Based on these optimization experiments, we also set a guideline for total sorting time of approximately two hours, to standardize the cytometer time which can impact cell viability, and thus also RNA yield and quality.

### Manual of procedures

To ensure consistent standardization across the BRITE study sites, the optimized and validated procedures were documented in detail in a Manual of Procedures (MOP). Specifically, the experimental protocols for cell processing, flow staining, instrument set up, data acquisition, cell sorting, and RNA storage were described within the MOP. Moreover, the MOP included information regarding the general BRITE study design, purpose, timelines, personnel contacts at the study sites, anticipated problems and solutions, etc. Thus, during the optimization phase, the MOP evolved into a central resource for each BRITE study site. The MOP was disseminated to the study sites and a revised digital copy was maintained in a cloud database with access for BRITE personnel.

## Discussion

Research studies with a multi-site flow cytometry design require considerable standardization procedures, careful selection of antibody clones and fluorophores, and matched cytometer instrument settings to enable accurate data comparisons [30]. To optimize the BRITE sarcoidosis study flow sorting procedures, we performed iterative optimization experiments to achieve a comparable resolution of the targeted CD4^+^ T cell subsets across the clinical sites. We followed an ordered process for optimization. The first step was identifying the protein markers that could resolve Th1, Th17, Th17.1 and Treg subpopulations. After the protein markers and potential flow cytometry antibodies were selected, the next step was designing a flow panel with attention to epitope abundance, fluorophore brightness, and anticipated resolution of the individual sites’ filter combinations. Following our initial testing, it became evident that flow panel optimization was necessary to enable comparable analysis of the cell populations across each site. Once an optimized flow panel was identified that enabled resolution of the targeted cell populations, standardization of the flow cytometer settings across each site was accomplished. Finally, we performed test flow cytometry sorting experiments to examine the success of the standardization process in the context of cell population resolution, sorting efficiency, and RNA sample quality across all study sites. Following these optimization steps, as outlined in **Fig 2A and 2B**, we documented the validated set of standardized protocols in the BRITE study MOP.

Given that these steps are time consuming, iterative, and require diligent planning and execution, the question arises, are they truly necessary for a multi-site study? Indeed, single cell sequencing approaches to analyze the peripheral blood compartment are becoming more common as technology enhances resolution, population identification, and transcriptome analysis. For example, current technologies such as CITE-seq enables simultaneous protein and RNA analysis at a single cell resolution [37], which could perhaps supplant the need for flow sorting coupled with bulk RNA-seq. However, these single-cell approaches have limitations as well. An obvious drawback of scRNA-seq vs. bulk RNA-seq is cost. The increased cost associated with scRNA-seq at this time makes it prohibitive for use with clinical studies analyzing multiple samples. Another limitation with single cell sequencing approaches, both multi-omics and single modality, is that only 500–5,000 cells per sample can be analyzed for a single sample. Cell hashing enables the analysis of more cells per sample [38], but cannot approach the millions of cells analyzed by bulk RNA-seq. Analyzing a relatively low number of cells (100’s-10,000’s) from the PBMC compartment is sufficient to identify major component cell types, such as B cells, T cells, macrophages, etc. However, identifying and performing differential expression analysis of additional rare cell types, such as Treg’s, across multiple human subject samples requires sampling larger numbers of cells. Performing this analysis with PBMCs using scRNA-seq approaches would require cost prohibitive sequencing and sample preparation. One methodology to get around this would be to pre-enrich a cell type such as T cells, to then more deeply analyze the subsets such as Th1 and Treg via scRNA-seq. Consequently, any pre-enrichment within a multi-site study structure would require optimization and standardization to ensure compatibility and accuracy of the data comparisons. Thus, depending on the breadth vs. focus of the research question, standardization efforts are an important component of clinical research study practices.

In summary, we focused on the following standardization and quality control steps, which we believe critical to performing a successful multi-site study: 1) Iterative optimization of a flow cytometry antibody panel that enables comparable resolution of target populations across all study sites; 2) Standardization of PMT voltages across sites using CST/rainbow beads with coordination of the same lot of beads across sites; 3) Implementation of a common flow cytometry template for gating and acquisition across sites; 4) Utilization of lyophilized flow cytometry staining reagents to reduce variability between study sites; 5) Generation of a standardized method of procedures (MOP) that details all experimental protocols, goals, troubleshooting, etc., to enable reproducibility between the study sites. The standardization steps outlined here can serve as resource for other clinical research studies with multiple sites employing flow cytometric sorting coupled with downstream NGS analysis.

## Supporting information

S1 Fig MFI comparison across sites based on CS&T bead voltages. These are the count plots used to compare voltages across sites.
(TIF)

S2 Fig MFI comparison across sites based on CS&T bead voltages. These voltages were used to adjust lasers at each site to maintain cross-site accuracy among cytometers.
(TIF)
