# Supplementary figures and images for: Standardization of flow cytometry and cell sorting to enable a transcriptomic analysis in a multi-site sarcoidosis study
Source: PLoS One. 2023 Mar 9;18(3):e0281210. doi: 10.1371/journal.pone.0281210 (PMC9997938; doi:10.1371/journal.pone.0281210)

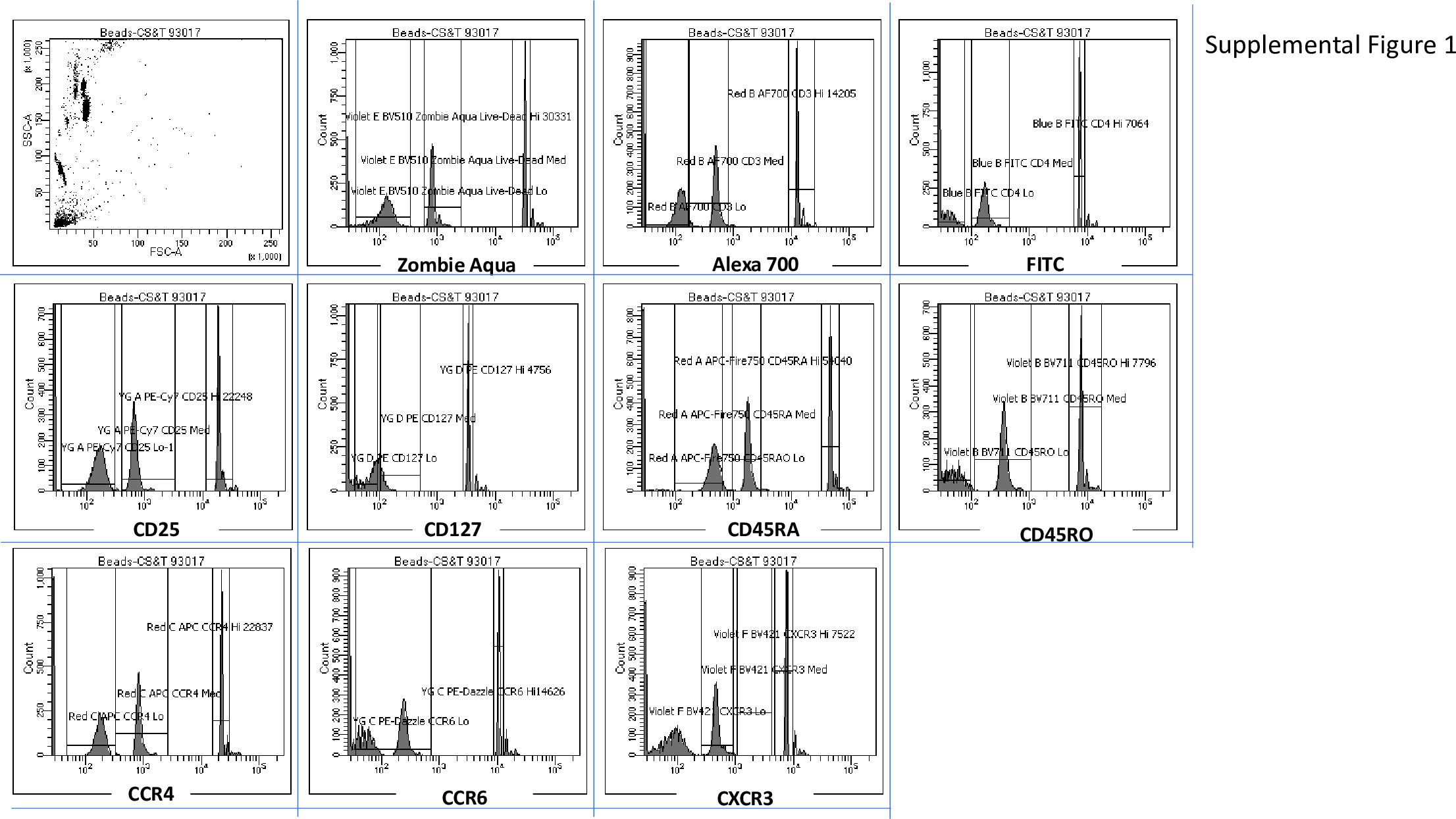

Supplement: S1 Fig — These are the count plots used to compare voltages across sites. (TIF) [file pone.0281210.s001.tif]

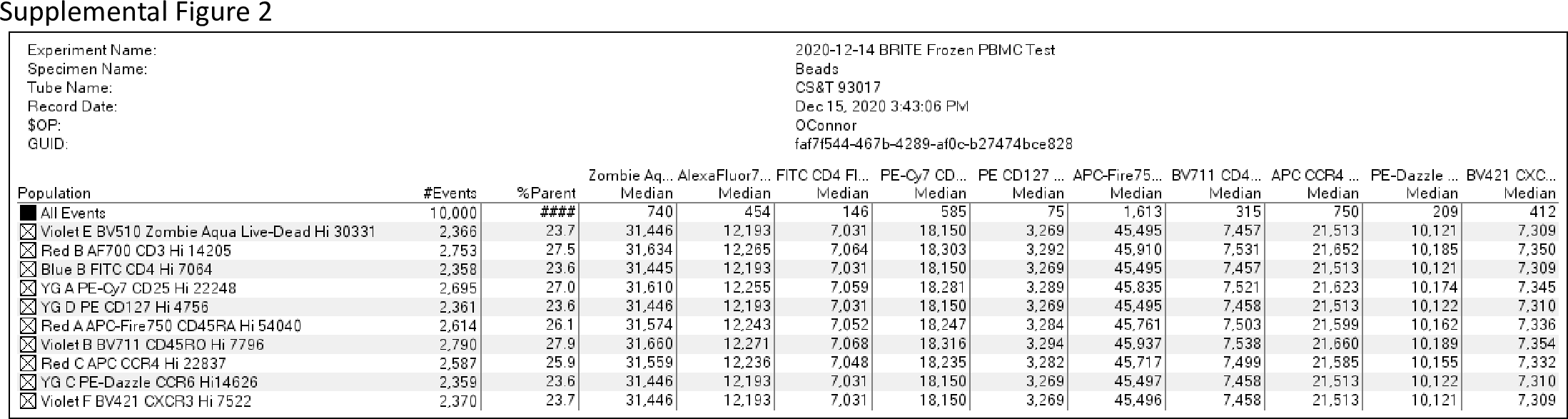

Supplement: S2 Fig — These voltages were used to adjust lasers at each site to maintain cross-site accuracy among cytometers. (TIF) [file pone.0281210.s002.tif]
